# Salvage Surgery After Non-Curative Endoscopic Submucosal Dissection for Early Colorectal Cancer: A Comprehensive Review

**DOI:** 10.3390/jcm14176343

**Published:** 2025-09-08

**Authors:** Felix Aigner, Christoph Skias, David Duller, Sebastian Wisiak, Karin Strohmeyer, Zoltan Horvath, Nicole Koter

**Affiliations:** Department of Surgery, St. John of God Hospital Graz, Marschallgasse 12, 8020 Graz, Austria; christoph.skias@bbgraz.at (C.S.); david.duller@bbgraz.at (D.D.); sebastian.wisiak@bbgraz.at (S.W.); karin.strohmeyer@bbgraz.at (K.S.); zoltan.horvath@bbgraz.at (Z.H.); nicole.koter@bbgraz.at (N.K.)

**Keywords:** salvage, surgery, submucosal, dissection

## Abstract

Endoscopic submucosal dissection (ESD) has emerged as a minimally invasive technique for treating early colorectal cancer (CRC), offering the potential for en bloc resection and precise histopathological assessment. However, when ESD results in non-curative outcomes—characterized by factors such as positive margins, deep submucosal invasion, or lymphovascular invasion—salvage surgery becomes a critical consideration. This review synthesizes current evidence on the indications, timing, surgical approaches, outcomes, and future directions of salvage surgery following non-curative ESD in early CRC.

## 1. Introduction

Colorectal cancer (CRC) remains the second leading cause of cancer-related death and the third most commonly diagnosed malignancy in Europe, with over 520,000 new cases and nearly 250,000 deaths annually as of 2023. Incidence rates vary across countries, but Western and Central Europe consistently show some of the highest burdens worldwide. Encouragingly, CRC mortality is declining in many high-income European nations due to improved screening programs, early detection, and advances in treatment.

Early-stage CRC (T1 tumors) has an excellent prognosis, with 5-year survival rates exceeding 90% if treated curatively. Endoscopic submucosal dissection (ESD), first developed in East Asia, has increasingly gained acceptance in Europe as a curative method for selected early lesions, particularly large, sessile, or non-polypoid neoplasms unsuitable for conventional EMR. ESGE guidelines recommend ESD for non-invasive lesions ≥20 mm or suspected superficial submucosal invasion in rectal tumors, as it allows en bloc resection and accurate histopathological assessment [1].

However, non-curative ESD—characterized by positive resection margins, deep submucosal invasion (>1000 μm), lymphovascular invasion, or poor differentiation—poses a substantial risk for residual disease or lymph node metastasis. Recent multicenter studies report non-curative resection rates ranging from 15% to 25% in routine ESD practice, necessitating careful post-procedural evaluation and, in many cases, oncologic salvage surgery [2]. En bloc and R0 resection rates, however, are still worse compared to transanal endoscopic microsurgery (TEM) for rectal neoplasms [3]. Although ESD achieves high en bloc resection rates—meaning the lesion is removed in one piece—its ability to ensure complete histological clearance (R0 resection) is often limited by challenges in achieving deep vertical margins, especially in fibrotic or invasive lesions. In contrast, transanal endoscopic microsurgery (TEM) allows for full-thickness excision under direct vision in an operative setting, often resulting in superior R0 resection rates for rectal neoplasms. Nevertheless, non-curative resections necessitate additional interventions to mitigate the risk of residual disease and metastasis.

In cases where ESD reveals high-risk histopathological features or incomplete resection—termed non-curative ESD—salvage surgery refers to a subsequent, typically elective, oncologic resection with lymphadenectomy, aimed at removing residual tumor and regional lymph nodes to reduce the risk of recurrence. This contrasts with primary colorectal surgery, as it is performed selectively following endoscopic therapy failure or uncertainty.

ESD is a minimally invasive, organ-preserving technique performed by advanced interventional gastroenterologists or colorectal endoscopists using a high-definition colonoscope equipped with specialized dissection knives. The procedure is carried out electively, under conscious or deep sedation and does not require an operating room setting. This contrasts with surgical options such as TAMIS (transanal minimally invasive surgery) or TEM, which are performed in the operating room under general anesthesia by colorectal surgeons.

In this context, salvage surgery plays a crucial role in ensuring long-term disease control. As ESD adoption rises across Europe, clinicians face the growing challenge of managing non-curative outcomes in a structured, evidence-based way. Given shifting epidemiology, evolving endoscopic techniques, and an urgent need for evidence-based protocols, this review aims to comprehensively evaluate and contextualize salvage surgery following non-curative ESD in early CRC.

## 2. Endoscopic Submucosal Dissection in Early Colorectal Cancer

### 2.1. Indications and Techniques

ESD is indicated for lesions with a low risk of lymph node metastasis, including superficial submucosal invasive cancers without lymphovascular invasion. The technique involves the precise dissection of the submucosal layer to achieve en bloc resection, allowing for thorough histopathological assessment. Proper staging of early colorectal cancer is paramount to avoid the problem of overstaging and unnecessary resection of large bowel.

### 2.2. Outcomes of ESD

Endoscopic submucosal dissection (ESD) in early colorectal cancer (CRC) demonstrates consistently high rates of en bloc and complete (R0) removal of lesions, yet also presents elevated complication risks compared to conventional methods. A pivotal multicenter study from Beijing analyzing 215 ESD cases reported an en bloc resection rate of 96.7% and overall R0 resection in the vast majority, with a low complication incidence of 4.7%, mainly minor bleeding and small perforations managed endoscopically [4]. Notably, non-curative resections occurred in 15.8% of patients—underscoring the need for vigilant histopathological evaluation post-resection.

In the context of colorectal ESD, R0 resection is defined as an en bloc resection with histologically negative lateral and vertical margins, indicating complete removal of the lesion without microscopic residual tumor. This distinction is critical for determining curative potential and guiding the need for additional intervention. In contrast, R1 resection refers to histologically positive margins, and Rx indicates unclear resection status.

Similarly, a retrospective cohort of 50 T1b CRC patients undergoing ESD found that 100% achieved en bloc resection and >80% had R0 margins, although vertical margin positivity persisted in ~18% [5]. Lymph node metastases were detected in 6%, particularly in cases with high-grade tumor budding and deep invasion (>1500 µm), identifying key risk factors for incomplete treatment and reinforcing the importance of specimen analysis for guiding further management [5].

Meta-analyses comparing ESD to endoscopic mucosal resection (EMR) consistently affirm ESD’s superiority in achieving en bloc and R0 resections, especially for non-pedunculated or large lesions. One systematic review across ~13,659 lesions showed en bloc resection rates of 90.5% for ESD versus 62.8% for EMR, and R0 in 82.1% vs. 92.0%—highlighting that while EMR may occasionally lead to R0 outcomes, ESD offers markedly fewer recurrences (~4% vs. >10%) [6].

The trade-off lies in procedure-related adverse events. Colorectal ESD carries a perforation rate of approximately 4–7%, commonly resolved via endoscopic clipping, with bleeding in 2–5% of cases [6]. Nevertheless, most complications are successfully managed without surgical escalation, supporting ESD’s overall safety when performed by experienced endoscopists. Real-world evidence emphasizes that meticulous peri-procedural planning and proper patient selection—taking into account lesion size, morphology, location, and operator expertise—are essential for optimizing outcomes.

To summarize, ESD achieves high curative resection rates for early CRC with low recurrence, albeit at the cost of modestly increased complications. Current literature consistently advocates for ESD in lesions >20 mm or those with high-risk features, provided adequate expertise and resources are available.

### 2.3. Defining Non-Curative ESD

Non-curative ESD is characterized by histopathological findings that indicate a higher risk of residual disease or metastasis, including the following:

Positive vertical or lateral margins;
  Deep submucosal invasion (>1000 μm);
  Lymphovascular invasion;
  Poorly differentiated histology.

These factors necessitate consideration of additional surgical intervention to achieve curative treatment.

## 3. Salvage Surgery: Indications and Timing

### 3.1. Criteria for Salvage Surgery

Salvage surgery after non-curative ESD is indicated based on specific histopathological findings that signify an increased risk of residual disease or lymph node metastasis. These include positive vertical resection margins, deep submucosal invasion (>1000 µm), lymphovascular invasion, poorly differentiated adenocarcinoma, and high-grade tumor budding [7].

Risk stratification tools like the eCura system for early gastric cancer [8] integrate these variables—along with tumor size—to classify patients into low-, intermediate-, and high-risk categories for lymph node metastasis: 2.5% in the low-risk group (0–1 points), 6.7% in the intermediate group (2–4 points), and up to 22.7% in the high-risk group (5–7 points). Patients in the intermediate- or high-risk categories are generally recommended to undergo salvage surgery, while low-risk patients—particularly the elderly or those with significant comorbidities—may be considered for intensive endoscopic surveillance. This system at least applies to the early gastric cancer.

Moreover, clinical decision-making must integrate patient-specific factors such as overall health status, comorbidities, life expectancy, and personal treatment preferences [9]. Shared decision-making in a multidisciplinary setting is essential to balance oncological benefit against surgical risk.

### 3.2. Clinical Decision-Making Framework

To guide treatment following non-curative ESD, we propose a structured clinical decision-making algorithm based on current evidence and guideline consensus. The following factors should be evaluated sequentially:

1. Patient-related factors
  Assess comorbidities, age, surgical risk (ASA score), and patient preference. For frail or elderly patients, intensive surveillance may be favored over surgery, especially in the low-risk group [10].
2. Multidisciplinary tumor board discussion
  Final treatment strategy should be individualized, involving input from surgical oncology, gastroenterology, pathology, and, when applicable, geriatrics and oncology.
3. Therapeutic options
  ○ High risk: Recommend salvage surgery with lymphadenectomy.
  ○ Intermediate risk: Surgery preferred; endoscopic reintervention may be considered in select cases.
  ○ Low risk: Consider surveillance with close endoscopic and radiologic follow-up.

This algorithm aims to translate the heterogeneity of histological findings and patient characteristics into a practical treatment pathway, balancing oncologic safety with invasiveness of intervention.

### 3.3. Timing of Surgery

Optimal timing for salvage surgery remains a subject of debate. Early intervention may reduce the risk of disease progression, but delayed surgery allows for patient recovery from ESD-related complications. Individualized assessment is crucial to determine the appropriate timing.

## 4. Surgical Techniques and Considerations

### 4.1. Surgical Approaches

Minimally invasive techniques dominate salvage surgery post-non-curative ESD. A recent cohort comparing conventional laparoscopic surgery (CLS), single-incision plus one-port (SILS+1), and three-port NOTES found all approaches had similar safety and oncologic outcomes [2,7,11,12]. However, SILS+1 and NOTES significantly reduced incision length, postoperative pain, and hospital stay while improving cosmetic satisfaction [11]. Emerging alternatives, such as colonoscopy-assisted laparoscopic wedge resection (CAL-WR) and combined endo-laparoscopic surgery (CELS), offer tailored solutions for lesions with deep invasion or fibrosis, expanding the paradigm of organ-sparing resections [11,13].

### 4.2. Intraoperative Challenges

Salvage surgery following non-curative ESD is technically demanding due to altered tissue characteristics. The presence of submucosal fibrosis and adhesions from prior ESD significantly complicates dissection planes. A retrospective study demonstrated that colorectal ESD in scarred or recurrent lesions resulted in increased perforation rates (32% vs. 4%) and extended operative times (117 vs. 61 min), driven by dense scar tissue and distorted anatomy [2,14,15,16,17]. Similar challenges are encountered during salvage laparoscopy; adhesions to the muscularis propria impede mobilization, raising the risk of enterotomies or bleeding. Surgeons must frequently adjust dissection strategies, utilize sharp rather than blunt techniques, and consider prophylactic adhesion barriers to reduce postoperative complications.

Another critical intraoperative hurdle is poor tissue perfusion secondary to prior interventions, which may impair healing. Utilization of intraoperative indocyanine green fluorescence angiography (ICG) can identify perfusion deficits: a prolonged DeltaT (time to fluorescence exceeding ~15.5 s) correlates with higher anastomotic leak risk [18]. Routine adoption of ICG-guided perfusion assessment has been associated with reduced leak rates and shorter hospitalization in salvage colorectal cases.

Sharp technical difficulty is common where fibrotic tissues adhere to surrounding structures. Multiport approaches (e.g., SILS+1, NOTES) that provide better exposure and instrument triangulation are particularly advantageous in these settings. Emerging techniques, such as CELS (combined endo-laparoscopic surgery), may offer organ-preserving options by targeting fibrosis-limited lesions through hybrid dissection, though they require experienced multidisciplinary teams.

Ultimately, successful salvage surgery demands meticulous preoperative planning, tailored intraoperative strategies—including traction techniques and fluorescence perfusion monitoring—and surgical expertise to navigate the fibrotic “hostile abdomen” created by prior ESD.

## 5. Oncological Outcomes Post-Salvage Surgery

### 5.1. Recurrence Rates

Local and distant recurrence after salvage surgery following non-curative ESD is rare but remains a critical measure of oncological success. Reported recurrence rates after non-curative colorectal ESD range from 0 to 16.2%, heavily influenced by completeness of resection and histopathologic risk factors [2,19,20,21]. In a pooled analysis of Japanese series, complete resections had local recurrence rates of 1.1–2.2%, whereas incomplete resections without further intervention showed much higher recurrence (7.5–25%) [20].

When salvage surgery is performed, recurrence dramatically decreases. For instance, in a long-term cohort of ESD followed by surgery for T1b lesions, no local or distant recurrences were observed among surgically treated patients, contrasting with recurrent events in non-surgical cases [5]. Similarly, a multicenter Japanese study of salvage surgery reported negligible recurrence after R0 resection in patients at intermediate to high risk from ESD [20].

Meta-analyses comparing ESD alone versus ESD plus surgery for high-risk lesions also support this trend. Local recurrence after ESD + surgery is near 0%, while non-surgical management yields recurrence rates up to 6% and metastatic recurrence in 5–6% of patients [20,22,23]. Moreover, the use of salvage ESD itself for residual or recurrent lesions shows promising long-term control—one prospective study reported zero recurrences at 60-month follow-up [17].

Despite these encouraging outcomes, risk remains in patients with positive vertical margins or lymphovascular invasion. A large European study of 632 en-bloc ESDs showed that incomplete resections had significantly higher recurrence (2.7% vs. 0.4%) compared to complete resections [2]. Factorial analysis indicated that the combination of two or more unfavorable histologic features increased residual tumor risk to over 50% and lymph node metastasis to 28% [2].

In summary, recurrence after salvage surgery post-non-curative ESD is exceedingly low, especially with R0 resection. However, individual histopathologic risk profiles remain critical; patients with multiple adverse factors benefit most from surgical intervention to minimize recurrence potential.

### 5.2. Survival Outcomes

Salvage surgery after non-curative ESD provides excellent long-term survival outcomes, comparable to primary radical surgery for early colorectal cancer. A Japanese retrospective cohort reported a 5-year overall survival (OS) rate of 95.5% and disease-specific survival (DSS) of nearly 100% among patients undergoing additional surgery post-non-curative ESD [20,22]. Similarly, a prospective series from the U.S. showed a 5-year OS of 93.6% and DSS of 99.6% after ESD with appropriate surgical follow-up (Table 1).

Comparative meta-analyses consistently demonstrate that patients receiving salvage surgery have superior OS and recurrence-free survival (RFS) compared to surveillance-only cohorts. One pooled analysis (n ≈ 2800) found that additional surgery was associated with nearly a threefold improvement in OS (OR = 2.95; 95% CI: 2.05–4.24) and more than doubled RFS (OR = 2.53; 95% CI: 1.38–4.62) [22]. Age stratification showed survival benefits across all age groups, including those ≥65 years [22].

In elderly patients (≥75 years), salvage surgery resulted in a 5-year DSS of 100%, compared to 96.3% in those managed non-operatively; however, overall survival was lower in the surveillance group (76.6% vs. 86.6%), reflecting comorbidity impacts [10]. This highlights the necessity for individualized decision-making, balancing oncologic gain against operative risk and patient frailty.

### 5.3. Adjustment for Confounding and Selection Bias

While multiple retrospective studies and meta-analyses suggest superior survival outcomes for patients undergoing salvage surgery after non-curative ESD, it is important to note that these comparisons are prone to selection bias. Patients chosen for surgery are often younger, have fewer comorbidities, and are generally fitter than those managed non-operatively. These factors independently contribute to improved overall survival and recurrence-free survival, regardless of oncologic intervention.

Most of the studies included in current literature are observational in nature, and only a minority employ statistical adjustments such as propensity score matching or multivariable regression modeling to account for confounding. As a result, the observed differences in survival between surgical and non-surgical cohorts must be interpreted with caution. Future prospective studies or randomized controlled trials are needed to validate the survival benefit of salvage surgery in risk-adjusted populations.

### 5.4. Key Takeaways

Surgery improves OS and RFS: The meta-analysis shows a threefold improvement in OS (OR 2.95) and more than doubled RFS (OR 2.53) with salvage surgery compared to surveillance alone [22].
  Excellent DSS after surgery: Cohorts combining complete ESD and salvage surgery report disease-specific survival rates near 99–100% at five years [24].
  Minimal recurrence after combined treatment: Japanese data show very low local recurrence (0.5%) in patients with curative salvage surgery [24].
  Similar DSS with or without surgery: In low- to intermediate-risk patients, DSS remained comparable (~93–94%) even without salvage surgery, but overall recurrence rates (~13%) were higher without surgery [7].

The evidence indicates that salvage surgery post-non-curative ESD significantly enhances overall and recurrence-free survival while achieving excellent disease-specific survival. Even though some low-risk patients may maintain favorable DSS without surgery, the added oncological safety of surgical intervention—particularly in higher-risk groups—is clear and clinically meaningful.

A summary of major studies comparing salvage surgery and non-surgical approaches is provided in Table 2 to facilitate comparison across outcomes and methodologies.

### 5.5. Lymph Node Metastasis: Risk and Controversies

The risk of lymph node metastasis (LNM) in patients undergoing non-curative ESD for early colorectal cancer remains a central and controversial issue in clinical decision-making. While the overall incidence of LNM in T1 colorectal cancers is relatively low—reported between 6–12%—the rate increases significantly in the presence of histopathological risk factors such as deep submucosal invasion (>1000 μm), lymphovascular invasion (LVI), poor differentiation, and tumor budding [20].

The eCura system, initially developed for early gastric cancer, may be adapted to colorectal cancer to stratify LNM risk post-ESD. Studies suggest that low eCura scores (0–1 points) are associated with <2% LNM risk, potentially justifying surveillance in carefully selected patients. However, high-risk scores correspond with LNM rates exceeding 10–15%, underscoring the recommendation for salvage surgery with lymphadenectomy.

Recent European multicenter analyses echo these findings, yet debates persist over the threshold of acceptable risk and the balance between overtreatment and oncological safety. Additionally, variations in pathological assessment—especially regarding LVI interpretation—further complicate uniform risk stratification. Efforts are ongoing to validate molecular and immunohistochemical markers (e.g., D2-40, CD34) to improve prediction of nodal involvement.

In summary, while salvage surgery remains the standard for patients with high LNM risk after non-curative ESD, emerging data may support a more nuanced, risk-adapted strategy, particularly in older or comorbid patients. Further prospective validation and standardized pathology reporting are needed to resolve ongoing controversies and refine clinical guidelines.

## 6. Morbidity and Quality of Life

### 6.1. Postoperative Complications

Salvage surgery after non-curative ESD in colorectal cancer is associated with postoperative morbidity rates similar to primary colorectal resections, but patient age, comorbidities, and fibrosis from prior ESD influence outcomes. A multicenter Austrian study reported major complications—Clavien–Dindo grade ≥III—in approximately 15–20% of rescue surgery cases, comparable to first-line surgery, with no excess mortality attributed specifically to prior ESD-induced changes [7].

Common complications include surgical-site infection (20–30%), anastomotic leak (up to 7%), and ileus or urinary retention. Anastomotic leaks often result from impaired perfusion and tension, with risk factors such as age, diabetes, obesity, and steroid use; they necessitate prompt intervention (e.g., drainage, re-operation, stoma creation) to reduce sepsis and mortality.

Elderly patients (≥80 years) experience severe complications at similar rates to younger cohorts, yet these complications significantly affect long-term survival—hazard ratio ~4 for cancer-specific mortality in older patients with major complications [25].

The presence of adhesions and fibrosis from prior ESD may prolong operative time and increase bleeding risk, but high-volume centers with multidisciplinary teams mitigate these hazards [26,27]. Application of enhanced recovery after surgery (ERAS) protocols has reduced complication rates, shortened hospital stays, and improved functional recovery in both elective and emergency colorectal surgery [28].

To minimize morbidity in salvage settings, meticulous preoperative assessment, adherence to ERAS principles, intraoperative perfusion monitoring (e.g., ICG fluorescence), and careful anastomotic technique are recommended, especially in frail or comorbid patients.

### 6.2. Long-Term Quality of Life

Long-term quality of life (QoL) following colorectal ESD remains favorable, proving advantageous over surgical alternatives. A multicenter cohort study in older patients (≥75 years) reported high 5-year overall survival (~95.8%) after ESD, with minimal decline in physical and mental health, indicating sustained QoL across frailty levels [10].

A systematic review of five-year outcomes after colorectal ESD showed low metachronous cancer rates (0.2–1.1%) and excellent disease-specific survival (98.6–100%), suggesting durable tumor control and minimal long-term distress [20]. Patients report minimal impact on daily life, with negligible long-term pain or dysfunction.

Comparative studies demonstrate that endoscopic treatment yields quicker recovery and less functional impairment than transabdominal surgery, without increasing cancer-related anxiety [29].

While rectal ESD can sometimes lead to transient bowel issues, overall satisfaction remains high, and most patients resume regular activities within months. Though Europe-specific QoL data remain somewhat limited, current evidence affirms that colorectal ESD maintains excellent long-term QoL and effectively addresses patient-centric outcomes.

Long-term QoL after salvage surgery following non-curative ESD is generally preserved, though certain functional and psychological domains warrant monitoring. Among organ-preserving resections (e.g., lower anterior resection), QoL scores equaled or surpassed those of abdominoperineal resection (with permanent stoma), though patients frequently experienced urgency and minor incontinence [30]. Notably, an analysis of older patients (>80 years) undergoing ESD ± salvage surgery reported ~90% 5-year survival with minimal decline in physical or psychological well-being, provided comorbidities were controlled [31].

Patients treated by salvage surgery typically recover baseline levels of daily functioning within 6–12 months, thanks to structured enhanced recovery (ERAS) protocols and pelvic rehabilitation [28]. However, subjective distress related to fear of recurrence and stoma use emerges in up to 30%, suggesting the value of psychosocial support and survivorship programs.

In summary, while salvage surgery can transiently affect bowel habits and emotional well-being, most patients achieve acceptable long-term QoL. Key enhancers include organ preservation, multidisciplinary perioperative.

## 7. Alternative and Adjunctive Therapies

Beyond salvage surgery, both adjunctive and less invasive alternatives play key roles in managing non-curative ESD cases.

### 7.1. Salvage Endoscopic Resection

Recent systematic reviews report en bloc resection rates of ~92% and R0 in 82% of residual or recurrent colorectal tumors managed endoscopically, with low recurrence (~2%) and minimal perforation (1–2%) or bleeding (~10%) risks [32]. Large multicenter cohorts demonstrate that salvage ESD or EMR affords effective local control, especially for small residual foci or superficial lesions, delaying or avoiding surgery in suitable patients.

### 7.2. Adjuvant Chemotherapy and Radiotherapy

Although formal evidence for adjuvant therapy post-ESD in early colorectal cancer (especially T1 lesions) is limited, extrapolations from stage II colorectal cancer guidelines suggest considering adjuvant chemotherapy for high-risk histology—such as lymphovascular invasion or poor differentiation. Consensus panels recommend discussing oxaliplatin-based regimens for patients with multiple risk factors following salvage surgery [33]. Additionally, in analogous scenarios (e.g., esophageal or gastric early cancers), chemoradiation after endoscopic resection has yielded survival outcomes comparable to surgery, while preserving organ function [34,35].

### 7.3. Multimodal and Hybrid Techniques

Hybrid techniques including combined endolaparoscopic surgery (CELS) are emerging as organ-conserving options. These enable resection of fibrotic residuals under direct visualization and may preserve bowel integrity. Early case series in gastric and rectal cancer suggest safety and feasibility in patients unsuitable for radical procedures.

### 7.4. Watch-And-Wait with Intentional Surveillance

For selected rectal early-stage lesions with complete endoscopic response or low-risk profiles, a watch-and-wait approach with strict follow-up may be an alternative. In such cohorts, salvage surgery remains effective upon regrowth, achieving high salvage R0 rates and comparable long-term outcomes to upfront resection.

In summary, salvage endoscopic reintervention is effective and safe for limited residual disease, adjuvant therapies may benefit high-risk cases, and hybrid or watchful strategies offer organ-preserving alternatives for well-selected patients.

## 8. Current Guidelines and Recommendations

Current international recommendations uniformly support additional intervention following non-curative ESD in early colorectal cancer. The European Society of Gastrointestinal Endoscopy (ESGE) defines curative resection as en bloc R0 resection of superficial submucosal (sm1/T1a) cancer without high-risk histology. Cases with deep invasion (>sm1), lymphovascular involvement, poor differentiation, or positive margins warrant multidisciplinary evaluation and generally recommend surgery or close surveillance in high-risk scenarios [1].

The Japanese Society for Cancer of the Colon and Rectum (JSCCR) guideline endorses the eCura scoring system to stratify lymph node metastasis risk post-ESD. Patients with intermediate or high eCura scores are advised to undergo colectomy with lymphadenectomy, while low-risk subjects may opt for surveillance [8].

The American Gastroenterological Association (AGA) and Multi-Society Task Force echo ESGE’s position: when histology demonstrates submucosal invasion or other high-risk features, referral for surgical resection is recommended, with endoscopic salvage reserved only for superficial positive margins. They emphasize referral of complex lesions to experienced centers [36].

Recent guidelines published in Gut and Liver reaffirm that surveillance alone is insufficient for non-curative resections. They report favorable long-term oncological outcomes when ESD is followed by recommended surgery for high-risk T1 CRC, highlighting that ESD now forms a foundational step in a ‘minimally invasive-first’ algorithm [37].

In practice, a multidisciplinary tumor board—including gastroenterologists, colorectal surgeons, pathologists, and oncologists—is required to determine personalized strategies. Decision-making should integrate histopathological risk (vertical margin, depth, lymphovascular invasion), patient comorbidities, and functional status. Growing interest in organ-sparing alternatives (e.g., salvage ESD, hybrid techniques) is evident in new guidelines, yet these are only advised when performed in centers with sufficient expertise and close follow-up protocols [38,39].

## 9. Conclusions

Salvage surgery after non-curative ESD for early colorectal cancer offers excellent oncological outcomes with low recurrence and high survival, particularly in patients with histological high-risk features. Minimally invasive techniques, individualized risk assessment, and multidisciplinary decision-making are essential. Emerging endoscopic and hybrid strategies expand treatment options, but surgery remains the gold standard for definitive curative intent in non-curative ESD cases.

### Limitations and Future Directions

The findings summarized in this review are primarily based on retrospective cohort studies and meta-analyses, which are subject to methodological limitations including selection bias, confounding, and inconsistent risk adjustment. Furthermore, the sample sizes in many surgical and endoscopic salvage cohorts are modest, often reflecting the low incidence of non-curative ESD cases. Variation in definitions of non-curativity, follow-up protocols, and institutional expertise also introduce heterogeneity that limits the generalizability of reported outcomes.

To advance the field, future prospective studies and multicenter registries are essential. These should incorporate standardized definitions, uniform histopathological assessment criteria, and long-term follow-up of oncological and functional outcomes. Propensity-matched or randomized controlled trials, although challenging to execute in this context, would be instrumental in validating current decision-making frameworks. International collaboration across high-volume ESD centers could provide the statistical power and external validity needed to optimize and personalize management strategies for patients following non-curative ESD.

## Figures and Tables

**Table 1:** Oncological outcomes—overall survival (OS), disease-specific survival (DSS), recurrence rate, and recurrence-free survival (RFS)—from recent studies on salvage surgery after non-curative colorectal ESD. CR: curative resection; OR: odds ratio; arrows indicate lower or higher rate.

Study & Cohort	5-Year OS (%)	5-Year DSS (%)	Recurrence Rate (%)	RFS/Recurrence-Free Survival
Meta-analysis (n ≈ 2800; surgery vs. surveillance) [22]	↑ OS (OR 2.95)	—	↓ Recurrence (OR 1.96)	↑ RFS (OR 2.53) significant advantage with surgery
Japanese multicenter ESD (CR + salvage surgery) [24]	93.6	99.6	Local 0.5	Comprehensive RFS; additional metastases rare
Nishizawa et al. (non-curative ESD, ±surgery) [20]	—	93 (no surgery) vs. 94 (with surgery)	~13% without surgery	—

**Table 2:** Summary of Key Studies on Salvage Surgery After Non-Curative ESD for Colorectal Cancer.

Author (Year)	Study Design	N (Patients)	Procedures Compared	Primary Outcomes	Key Findings
Nishizawa et al. [20]	Systematic Review	1600+	ESD ± Surgery	Recurrence, DSS	DSS 93–94%; ~13% recurrence without surgery
Ohata et al. [24]	Prospective Cohort (Japan)	1000+	ESD + Surgery	5-year OS, Recurrence	OS 93.6%, DSS 99.6%, Local recurrence 0.5% without surgery
Jia et al. [22]	Meta-analysis	~3500	Surgery vs. Surveillance	OS, RFS	OS: OR 2.95, RFS: OR 2.53 in favor of surgery
Dell’Anna et al. [16]	Retrospective Multicenter	178	Salvage ESD for fibrotic lesions	Local recurrence	3.3% local recurrence

## Data Availability

Data supporting reported results can be found in the National Library of Medicine.

## Abbreviations

The following abbreviations are used in this manuscript:

ESD	Endoscopic submucosal dissection
CRC	Colorectal cancer
TEM	Transanal endoscopic microsurgery
CELS	Combined endo-laparoscopic surgery
CLS	Conventional laparoscopic surgery
NOTES	Natural Orifice Transluminal Endoscopic Surgery
SILS	Single-incision laparoscopic surgery
CAL-WR	Colonoscopy-assisted laparoscopic wedge resection
ICG	Indocyanine green fluorescence
OS	Overall survival
DSS	Disease-specific survival
RFS	Recurrence-free survival
OR	Odds ratio
ERAS	Enhanced recovery after surgery
QoL	Quality of life
EMR	Endoscopic mucosal resection
